# In Their Own Words: A Qualitative Investigation of the Factors Influencing Maternal Postpartum Functioning in the United States

**DOI:** 10.3390/ijerph17176021

**Published:** 2020-08-19

**Authors:** Ariana M. Albanese, Pamela A. Geller, Jackson M. Steinkamp, Jennifer L. Barkin

**Affiliations:** 1 Department of Psychology, Drexel University, Philadelphia, PA 19104, USA; pg27@drexel.edu; 2 Hospital of the University of Pennsylvania, Philadelphia, PA 19104, USA; jackson.steinkamp@pennmedicine.upenn.edu; 3 Mercer University School of Medicine Department of Community Medicine, Macon, GA 31207, USA; barkin_jl@mercer.edu

**Keywords:** maternal postpartum functioning, maternal–child health, women’s health

## Abstract

During the first twelve months postpartum, infants require intensive care and mothers are susceptible to physical and mental health concerns as they undergo a period of tremendous psychological and physiological adjustment. The mother’s level of postpartum functioning not only impacts her experience as a mother but also the infant and family unit. However, efforts to bolster functioning are lacking, and previous literature has identified a gap between what experts recommend and what mothers desire during the postpartum period. To address this, we conducted structured interviews with a diverse sample of 30 postpartum mothers to identify factors that mothers report are most influential to their postpartum functioning. In total, we identified 23 clinically actionable factors, all of which are backed by existing literature. In addition to an in-depth presentation of the qualitative findings, we also present a heat map to visualize the relevance of these factors to each of seven established domains of maternal functioning. Lastly, based on our findings, we offer a taxonomy of interventional strategies that could bolster maternal functioning during this critical period.

## 1. Introduction

Level of functioning, one’s ability to perform the activities and roles required to maintain health and wellbeing, is of high importance during the postpartum year for individuals who have given birth. It is important to note, at the outset, that we will use the term “mothers” in this article to refer to these individuals, but this topic is relevant to anyone who can become pregnant, such as trans men and non-gender binary individuals. Level of maternal functioning is an important determinant of infant health as mothers are generally the principal performers of infant care [1], and these early interactions with key caretakers can influence later development [2]. Additionally, maternal postpartum functioning plays an important role in maternal wellbeing as the postpartum year is a time of increased vulnerability to physical health concerns [3] and is also the time period in which mothers are most likely to develop depression and anxiety compared to any other time in their life [4]. These health concerns can often worsen due to the many barriers to postpartum healthcare visit attendance [5] as well as the neglect of self-care commonly experienced by postpartum mothers [6]. Therefore, assuring that mothers are achieving optimal functioning during this time period is an important health goal for both the mother and the child.

Despite the recognition that the postpartum year is a vulnerable and important time, existing approaches to support postpartum mothers are lacking, especially in the United States. More commonly available initiatives, such as prenatal education, often fail to properly prepare mothers for postpartum adjustment [7]. Importantly, there is also a gap between what healthcare providers believe postpartum mothers require to promote wellbeing during the postpartum period and what is desired by the postpartum mothers themselves [8,9,10]. A recent review also called for better clarification of the dimensions of health which promote holistic maternal wellbeing and functioning in the postpartum year, beyond commonly researched topics such as depression and breastfeeding [11].

With the creation of the Barkin Index of Maternal Functioning, the field now possesses a patient-centered means of assessing functioning in the postpartum period across seven domains [12,13,14]. However, before we can improve upon existing interventions to bolster maternal functioning during the postpartum period, we must first clarify which specific factors help and hinder maternal functioning during this time period.

To address this issue using a patient-centered approach, we administered structured interviews to a diverse sample of postpartum mothers (*n* = 30). In order to identify the key factors that influence a mother’s assessment of her functional capability, we used the Gist and Mitchell (1992) cyclical model of self-efficacy formation, which identified the following as the key processes preceding one’s assessment of capability: an analysis of task requirements, an attributional analysis of experience, and an assessment of personal and situational resources and constraints [15]. Participants were thus asked: (1) what is required to function well, (2) what has helped or hindered function in the past, and (3) how equipped does one feel to function well in their current personal and situational context, within each of the seven established domains of maternal functioning [12]. This allowed us to obtain a broad understanding of what mothers have observed is necessary to function well in the postpartum period.

## 2. Materials and Methods

As mentioned, the present study examined three key elements preceding one’s assessment of capability within each of the seven established domains of maternal postpartum functioning. These domains are as follows: self-care, infant care, mother–child interaction, psychological wellbeing, social support, responsibility management, and adjustment [12]. The data collection was conducted via individual interviews with mothers in the postpartum period, which were subsequently coded for thematic content. Several key indices, namely, basic sociodemographic information, level of stress, level of maternal functioning, and level of depression and anxiety, were also collected through self-report measure after these interviews. Details on participants, design, procedures, and analyses are provided below.

Eligible participants were individuals who had given birth in the past twelve months to a singleton infant born at term without any chronic medical conditions, and who were the primary caretaker for that infant. Eligible individuals were at least 18 years of age, and both literate and fluent in English. Individuals were considered ineligible if they failed to pass a 6-Item Cognitive Screen which tests orientation and recall [16], indicating that they were not properly oriented and prepared for an extensive interview.

**Figure 1:**
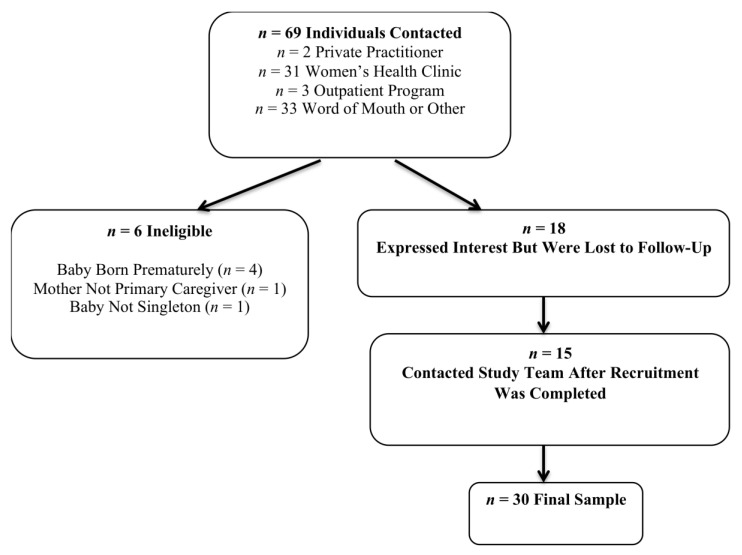
Participant recruitment, enrollment, and flow information.

Recruitment occurred between March and September 2019 in a large Northeastern US city. Participants were recruited via flyers posted in community establishments catering to new mothers and by word of mouth. In-person recruitment was also held in one clinic and one outpatient program targeting women’s health. Additionally, the study was advertised in the office of a private practice provider who treats postpartum women. See Figure 1 for participant flow information. Each participant was compensated with a $40 gift card upon completion of the interview and brief self-report assessment battery.

All participants provided informed consent for inclusion in the study before participation, and all interested individuals passed the cognitive screen. Participants were informed that participation was entirely voluntary and that they had the right to withdraw from the study at any time. The study visit was conducted in a private office space, participants were assured of anonymity and confidentiality, no identifying information was collected, and all responses were kept anonymous. All concerns were clearly outlined in the consent form and explained to all eligible participants. The study was conducted in accordance with the Declaration of Helsinki, and the protocol was approved by the Institutional Review Board of Drexel University on 22 March 2019 (IRB ID: 1812006846).

A structured interview designed specifically for the current study was conducted with each participant in order to gather qualitative data concerning key elements impacting maternal functioning in the postpartum period. An interview guide appears in Appendix A. As mentioned, for each of the seven established domains of maternal functioning in the postpartum period (self-care, infant care, mother–child interaction, psychological wellbeing, social support, responsibility management, and adjustment) [12] participants were asked about three key elements which impact one’s assessment of their functional capability within that domain, specifically:

1. An analysis of task requirements, or what is required to function well in that domain;
2. An attributional analysis of experience, or what has helped or hindered function in that domain in the past;
3. An assessment of situational resources and constraints, or how equipped one feels to function well in that domain in their current personal and situational context.

The study staff member conducting the interview (AA) made efforts to distribute interview times evenly across all domains. The order in which domains were discussed was varied as follows: the domains were ordered, numbered, and with each subsequent interview, the order of discussion of the domains was shifted. This was intended to guard against a tendency to spend more time on the first domains assessed (e.g., as the participant acclimates to the study questions/format).

In addition to the qualitative interview, five self-report measures were administered. These measures included a 21-item sociodemographic questionnaire created for the current study (used to collect information regarding participants’ age, race/ethnicity, gender, sex, religious identity, education level, household income, employment status, couple or relationship status and length of relationship, partner gender (if partnered), infant’s sex, infant’s age (in months), presence of additional caretakers for the infant, how many children they have, and the participant’s assessment of their own mother’s self-efficacy). The Perceived Stress Scale-10 [17], the Barkin Index of Maternal Functioning [12], and the Hospital Anxiety and Depression Scale [18] were also administered. A more detailed description of these measures is provided below.

The Perceived Stress Scale-10 [17] is a shortened version of a measure of perceived stress. The measure contains 10 items that inquire about thoughts and feelings concerning the degree to which participants find their current life situation uncontrollable, unpredictable, and stressful. On a 5-point Likert scale (ranging from 0 = never to 4 = very often), the participants answer how often they have thought or felt a certain way. A higher score indicates a higher amount of perceived stress. Adequate internal consistency was achieved for this measure (Cronbach’s α = 0.91).

The Barkin Index of Maternal Functioning [12] is a measure of maternal functioning in the first 12 months postpartum. The measure contains 20 items that prompt the participant to respond on a 7-point Likert scale (ranging from 0 = strongly disagree to 6 = strongly agree) to a series of statements regarding seven functional areas of new motherhood (self-care, infant care, mother–child interaction, psychological wellbeing, social support, responsibility management, and adjustment). A higher score indicates a higher degree of functioning. Adequate internal consistency was achieved for this measure (Cronbach’s α = 0.80).

The Hospital Anxiety and Depression Scale is a 14-item scale used to identify symptoms of anxiety and depression. Participants use a 4-point Likert scale to indicate the frequency or severity of symptoms, with higher scores indicating more severe symptomatology [18]. Two subscale scores are derived (one for depressive symptoms and one for anxious symptoms) and scores of 8 or higher on either subscale are considered elevated. Adequate internal consistency was achieved for both the anxiety subscale of this measure (Cronbach’s α = 0.86) as well as the depression subscale of this measure (Cronbach’s α = 0.73). It should be noted that while the Edinburgh Postnatal Depression Scale [19] is most commonly used to identify depressive symptoms in the postpartum population, we chose to administer the Hospital Anxiety and Depression Scale as this allowed for formal measurement of both anxiety and depression in one brief 10-item scale, thus allowing for the measurement of both constructs while reducing participant burden.

Following data collection, all interviews were transcribed and thematically coded utilizing inductive content analysis by two study team members (AA and JS) using QSR International’s NVivo 12 qualitative data analysis software [20]. This coding allowed us to extract the qualitative themes that represented factors that mothers perceived as impacting their functioning. Codes were not decided a priori but were iteratively developed as the coders independently reviewed the transcripts. Content analysis was utilized, and guidelines for engaging in inductive content analysis were followed [21]. The specific steps taken were as follows: First, the entire corpus of transcripts was independently reviewed by the two coders during an “open coding” phase, in which all identified themes were independently labeled. Of note, thematic saturation was achieved for both coders after independent review of ten interviews. Second, the coders met and discussed their identified themes, integrating and abstracting these labeled themes into a hierarchical coding scheme. Third, the coders engaged in several rounds of “testing” the coding scheme on a subset of interviews, meeting in between rounds of “testing” to discuss any discrepancies and further refine the coding scheme. Fourth, the coders independently reviewed and coded the entire corpus of interviews using the updated coding scheme. A concordance of 0.71 was achieved between coders after independent review. One of the coders (AA) reviewed the entire corpus a final time following independent review, resolving any remaining discrepancies with frequent consultation from the second coder (JS), until a final reorganization of codes was achieved. It should be noted that discrepancies were resolved through discussion between AA and JS until consensus was reached.

Additionally, a heat map of the occurrence of factors across the seven domains of functioning was created to better visualize the relevance of these factors to the seven domains of maternal functioning. The 23 uncovered factors influencing maternal functioning are represented in rows in the diagram, and domains of functioning are represented in columns. Each cell (the cross of the factor and domain) is color-coded by the number of participants (out of *n* = 30), who discussed that factor in that domain. The darker the color of the cell, the greater the number of participants who discussed that factor (a legend for interpreting the color-coding appears at right of the figure). Lastly, descriptive statistics were calculated for all self-report data.

## 3. Results

### 3.1. Participant Characteristics

A sociodemographically diverse sample of 30 mothers was recruited (see Table 1). Though the majority of study participants were White, nearly half (46.7%; *n* = 14) were non-White, and the study sample included individuals representing several racial and ethnic identities. There was also fairly even representation across levels of education and religious affiliations in the sample. The sample had similar representation between those who were primiparous (56.7%, *n* = 17) and those who were multiparous (43.3%; *n* = 13). The distribution of household income in the sample was almost bimodal, with 23% (*n* = 7) in the top bracket at more than $150,000 per year estimated household income, and 30% (*n* = 9) in the lowest bracket at less than $25,000 per year estimated household income.

The relative diversity of the sample is likely a result of utilizing multiple recruitment methods, as different recruitment venues seemed to inadvertently target different demographic groups. For example, of the 13 participants recruited at the women’s health clinic, the majority were Black or African American (*n* = 10, 77%) and their annual household income estimated at less than $25,000 (*n* = 8, 62%). However, the 17 participants who were not recruited at the women’s health clinic were majority White (*n* = 16, 94%), and the most commonly estimated household income bracket was more than $150,000 (*n* = 7, 41%). While we did not note major differences in themes across sociodemographic groups, the interviews performed with the women’s health clinic subsample tended to be shorter on average with a mean length of 42 min (±8.8) than the subsample that was not recruited in this context, which had a mean interview length of 52 min (±15). It is possible that this is due to the fact that individuals recruited at the clinic did not anticipate they would be participating in the study visit, and thus felt more rushed to finish, while those who were recruited in other contexts were able to schedule their study visit ahead of time and may have felt they could complete the visit at a more leisurely pace.

The reported level of anxiety in this sample was high, with 40% of participants (*n* = 12) scoring in the clinical or borderline clinical range on the Hospital Anxiety and Depression Scale―Anxiety Subscale, while the estimated population rate for elevated postpartum anxiety is 11–17% [22]. The reported level of depression in the sample was about equal to the postpartum population rate [23], with 13% of the sample scoring in the clinical or borderline clinical range in depressive symptoms. The mean score on the Perceived Stress Scale-10 (13.37) indicated typical levels of stress in the sample as it was slightly lower than the population norm for women (13.7). The mean score on the Barkin Index of Maternal Functioning was 97.9 (SD = 10.66), indicating ideal functioning in the overall group (as scores to the right of 80 on a number line are considered within the realm of ideal functioning).

### 3.2. Factors Impacting Maternal Postpartum Functioning: Qualitative Themes

Twenty-three factors impacting maternal function were gleaned from the interviews. Each of these has precedent in the literature. Table 2 displays these factors, relevant citations, and example quotes. All factors are also discussed in detail below, where they appear in alphabetical order. Of note, where relevant, reference is made to the table to provide an example quote. This is intended to guard against duplication of information. However, in instances in which the inclusion of another quote was deemed helpful to illustrate the phenomenon being discussed, an additional quote is included in the descriptive paragraphs below.

#### 3.2.1. Accurate Locus of Control, Limiting Inappropriate Self-Blame

Participants frequently discussed perceived control (*n* = 12, 40%). They acknowledged that certain elements of their child’s wellbeing, whether positive (e.g., easygoing temperament) or negative (e.g., difficulty feeding) are not solely the result of their actions as a mother. However, despite this knowledge, participants noted that they often interpret negative experiences as if they were a direct result of their parenting. This, in turn, detracted from their sense of themselves as a parent and made them “feel like a failure”. ID 17 encapsulated this self-blame, stating:

“*[Moms who were struggling to breastfeed] were doing this triple pump thing where you feed the baby and pump, and then you give it a bottle, and then you do this and you do that, like ten times a day. And they felt like they were failing because it wasn’t working. And I feel like that was kind of the most eye-opening thing to me that they were spending all this time dedicated to their babies and still felt like they were doing a bad job*.”

This interpretation bias echoes previous literature, which has linked excessive maternal self-blame to negative outcomes such as depression [24,25].

#### 3.2.2. Adaptive Attitude towards Learning and Adjustment

Three specific aspects of one’s attitude towards learning and adjustment were discussed by participants (*n* = 28, 93%): (1) the ability to “take the long view” and be patient with the day-to-day ups and downs of parenting, (2) comfort with trying new things, and (3) cognitive flexibility and willingness to accept when plans change or expectations are not met.

While all postpartum mothers must contend with the challenge of engaging in new activities for the first time, participants described experiencing different levels of comfort with trying new things. Some participants described experiencing an easy willingness to try new things, while others experienced anxiety at this prospect, and had to engage in self-counseling to work up the motivation to try something new (see Table 2 for example quote).

Cognitive flexibility was a particularly salient theme, which was first important for reducing the distress participants experienced when their expectations were not met. Previous work has described the gap between expectations and reality in motherhood, and how the mismatch of expectation to reality can lead to self-deprecation and depression for mothers [28]. ID 1 described the difficulty she faced in adjusting her expectations, stating:

“*at the time, … it seemed very important to feed him a particular way and I was attached to ultimately being able to do that. And it’s hard when you’re that emotional and hormonal and sleep-deprived to … realign your expectations so dramatically from what you thought they would be.*”

Additionally, cognitive flexibility was important for participants to creatively improvise new ways to accomplish their goals. For example, ID 4 described incorporating her older daughter into her infant care routine in order to satisfy the infant’s need for care simultaneously with the older child’s need for attention.

#### 3.2.3. Bond with Baby

Participants’ reported experience (*n* = 16, 53%) supported the previously identified importance of the mother–infant bond in determining maternal outcomes [30,31]. Specifically, participants noted that it was important for them to protect bonding time with the baby (setting aside time in their schedule), and to be mindfully present during that bonding time (not multitasking, not scrolling on one’s cell phone, not being lost in one’s thoughts). As ID 24 described, active mindful engagement in infant bonding, “playing with him, rolling on the floor, pretty much just working to make him smile or whatever it may be. Talking to him and teaching him things.” Participants noted that this dedicated bonding time not only helped them to learn more about their infants (see Table 2 for example quote), but also improved the degree of enjoyment they experienced during interactions with their infants, as ID 25 described, “It takes a little bit of effort to sit down and engage for me, but it’s been great. I really like it. I really like playing with her.”

#### 3.2.4. Child Temperament

In line with literature linking infant colic to negative maternal outcomes such as poor psychological adjustment [32], participants reported baby’s mood as an important factor influencing their distress and perceptions of their parental functioning (*n* = 27, 90%). As described by ID 21 (see Table 2 for example quote), it is very upsetting for mothers to endure the crying of their infants for long periods of time, and failed attempts to stop the crying can result in feelings of inadequacy. However, if mothers are able to remind themselves that it is okay for an infant to cry, the associated degree of distress and feelings of inadequacy can be lessened. This point is described by ID 23:

“*I think me being calm and knowing that he’s okay and if he cries, just going through the checklist. Did he eat, did he sleep, did he poop? If he had all his things, then maybe he needs one extra feed or a little bit more sleep or whatever. But … if he did everything today, then he is good.*”

#### 3.2.5. Emotion Regulation

Participants reported that emotion regulation played an important role in their postpartum experience (*n* = 29, 97%). Many participants referred to effective emotion regulation as “having patience” and being able to stay calm in the face of challenging situations. Some mothers reported that negative emotions, such as anxiety, frustration, or feelings of failure, got in the way of the successful completion of postpartum activities. However, others noted that they effectively engaged in cognitive and mindfulness-based strategies to regulate their emotions, which have been proven to be effective in improving postpartum emotional health [33]. For example, ID 18 described utilizing mindfulness-based approaches (see Table 2 for example quote).

Additionally, several mothers described engaging in religious practices to effectively regulate their emotions, which has been established as an effective coping tool for postpartum mothers [34]. As ID 7 described, “I literally just pray, definitely pray. Because if I don’t, it would be something else … That calms me down. Even if it’s outside the kids, praying calms me down.”

#### 3.2.6. Financial and Material Resources

Low-income status is an established risk factor for poor postpartum outcomes such as depression [23,36], and previous qualitative work has revealed how poverty can be self-assessed as a maternal failing [35]. Participants described financial and material resources as paramount (*n* = 21, 70%) not only given need to pay for the apparatus required to raise a baby (diapers, clothes, feeding supplies) but also because money is often necessary to acquire support such as childcare, specialist help (e.g., lactation consultation), or assistance with house cleaning or meal preparation. Participants indicated that having the financial resources to pay for assistance when necessary was key to successful completion of all the responsibilities inherent to motherhood while also attending to one’s own needs (see Table 2 for example quote).

#### 3.2.7. Gaining Firsthand Experience with Parenting Activities

Participants reported that they felt more successful and capable as parents as they gained more experience parenting (*n* = 24, 80%). Some participants reported that prior experience taking care of infants (such as younger siblings or cousins) helped them, while others reported that the experience of parenting their particular infant was the most beneficial, as every child is different (see Table 2 for example quote). This finding supports previous literature. First, it demonstrates that more experience parenting is associated with parenting competence and confidence [37,42]. Second, it indicates that competence in caring for an individual infant improves by virtue of spending more time caring for that infant, regardless of whether one has historical infant care experience [38].

#### 3.2.8. Giving Oneself Credit for Successes

Participants indicated that it was important to take the opportunity to note their successes in parenting (*n* = 16, 53%). Despite apparent cultural norms that discourage maternal engagement in lauding their own performance (see Table 2 for example quote), participants indicated that when they did take the time to “savor” their successes, it served to provide them with both the comfort that their child was appropriately cared for (ID 1 referred to this as her baby “being on track”) as well as the positive feeling of pride in their accomplishments. As ID 14 described her accomplishment of arranging a day at the museum with her daughter:

“*she was having fun, so I was having fun. There wasn’t no crying, there was nothing, she was laughing the whole time. She was having fun, and I felt proud of myself because I did everything, and I set everything up, and it was just- … just everything was nice. I was happy*.”

This finding also supports previous work which has linked self-criticism with negative postpartum outcomes and self-esteem with positive outcomes [40,41].

#### 3.2.9. Insufficient Time for Task Demands

Participants frequently lamented a lack of time to complete all the tasks inherent to their daily responsibilities (*n* = 26, 87%; see Table 2 for example quote). The transition to parenthood is rife with new task demands and role requirements, which can elicit stress from parents [84] especially if they lack social support to help them complete all that is expected of them. ID 17 described this as “the lack of time. When you feel like there’s not enough hours in the day.” This was particularly salient for mothers who worked (such as the participant quoted in Table 2), who experienced additional time pressure to complete both work and domestic tasks throughout the course of the day.

#### 3.2.10. Internal Aspects of Engagement with Social Support

Five key internal factors were reported by participants that allowed them to effectively utilize their social support network (*n* = 27, 90%; see Table 2 for example quotes). These included: (1) an ability to be vulnerable with social support (i.e., an ability to be open and honest about one’s thoughts, emotions, and experiences); (2) an ability to “let go” and trust others to take care of the baby so they could take a break or attend to a non-infant task; (3) comfort with asking for and accepting help from social support providers; (4) their skill in effectively communicating with members of this social support network; and (5) recognizing the limit of what they can handle independently and knowing when they ought to employ the help of their social network.

#### 3.2.11. Keeping Baby in a Routine

Participants identified the establishment and maintenance of routines for their children as allowing for them to regain some sense of control (*n* = 14, 47%). Mothers reported feeling relieved distress due to the predictability of a bedtime or feeding routine, which granted them a degree of certainty about how their day would go. For example, as opposed to approaching each bedtime with the fear that they would be up for hours, the establishment of a routine allowed them to feel confident that parenting activities would go as planned. Previous work has also found routines to be beneficial, particularly as they relate to sleeping schedules [51].

#### 3.2.12. Knowledge Access

Access to knowledge about parenting and early motherhood was identified as important by participants (*n* = 26, 87%). This knowledge took several forms, and preferences for different forms of knowledge varied among participants and across contexts. This is consistent with prior working reporting that mothers seek information from multiple sources [85].

First, participants identified knowledge as disseminated through educational resources (e.g., books, medical literature, consultation with professionals such as pediatricians and lactation consultants) as a helpful source of guidance. As ID 21 explained:

“*for me, it’s like what the data says, more I guess because of my background and my own child. But it’s everything data, data, data. And people can give you their opinions. But I’d like to see what the research actually says. Reading a great new book called Crib Sheet, which is like the data-driven guide to more relaxed parenting*.”

Previous literature has linked this formal education with positive outcomes for parents [52,53].

On the other hand, some participants preferred the knowledge stemming from experience, and thus relied on asking questions of peers, such as members of dedicated mothers’ groups or experienced mothers with firsthand knowledge of parenting. As ID 19 explained:

“*having the opinion from other people. Maybe what people usually do. That’s also helpful to kind of compare what you’re doing with what other people are doing. Not in a negative way but just to have somebody else’s experience so that you can kind of know if you’re doing good or not*.”

Previous literature has also discussed the value of access to experienced individuals, of whom mothers can ask questions and learn what other mothers have found to be helpful in their firsthand experience [54].

Interestingly, participants expressed differing opinions on use of the internet for information. Several participants stated that they used internet-based applications for information, while others were wary of the internet, stating that there is “bad information” there. Other qualitative inquiry has found a similar wariness of the internet as a source of information [86].

#### 3.2.13. Maintaining Aspects of Life Outside of Parenting

While motherhood is a fulfilling experience for many, participants identified the importance of continuing to engage in activities outside of parenting (*n* = 14, 47%). For example, some participants reported feeling a sense of loss due to decreased participation in activities outside of the home. For instance, several participants who experienced alterations in their work schedule stated that they no longer felt “*productive.*” As ID 28 described:

“*I was a workaholic before this, and it’s really weird to not feel like I’ve contributed or really done something in a day, and I need to be more okay with taking care of the baby as doing something, but you just feel like you’re sitting on a couch all day long*.”

Accordingly, several participants reported working as a positive experience because it was an important part of their pre-motherhood identity that they were able to maintain after giving birth. ID 1 even referred to work as “self-care because I’m not doing it for my child. And at some point, I get to leave the house and have a life outside of my family.” This echoes previous work examining the sense of loss associated with transitioning from working outside the home to fulltime caretaking [55].

Participants also described the importance of continuing to socially engage outside the home (see Table 2 for example quote). This is in line with previous work, which describes the negative impact of the social isolation that is often accompanies the postpartum period [56].

#### 3.2.14. Mother’s Self-Knowledge

Several participants described the importance of “knowing yourself” (*n* = 20, 67%). This self-knowledge frequently centered on topics relevant to self-care and psychological health, and participants identified a knowledge of what coping strategies work for them and what self-care activities bring them the most emotional nourishment as the primary step which must be taken before effective emotion regulation strategies or self-care plans can be put into action. Previous work has identified self-knowledge as key to processes such as self-actualization [57], relevant to the role and identity transition that occurs postpartum.

#### 3.2.15. Physical Home Environment

Participants discussed the role of the stability, safety, and comfort of their home environment in their postpartum experience and their maternal functioning (*n* = 15, 50%). In more extreme cases, the stability of the participant’s home was threatened (see Table 2 for example quote), which was a major stressor impacting their ability to parent. Other participants discussed having limited ability to spend time outside of their homes due to living in unsafe neighborhoods. This limited what they could do for self-care and the activities they could do with their children. As ID 16 described, “this is the only place that’s safe because you go right in the house and run and lock the door … You can’t really do everything.” In less extreme cases, loud housemates decreased the level of comfort in the home environment, and posed some obstacle to engaging in quieter and more meditative activities. Additionally, several mothers reported that seasonal weather (extreme heat in summer or cold in winter) occasionally made it difficult for them to engage in activities like walks with their infant. This finding reifies the role that physical environment plays on maternal wellbeing [58].

#### 3.2.16. Prioritization of Self-Care

Participants also indicated that the degree to which self-care is prioritized by mothers is important (*n* = 24, 80%). Reiterating themes uncovered in previous qualitative work [59], some participants stated that they felt guilty for spending time on themselves away from their child. However, other participants expressed more of a “buy in” to the idea that a neglect of self-care is destructive to one’s wellbeing, and they accordingly prioritized taking care of themselves. As ID 30 stated:

“*I watched other people kind of struggle with that and feel like they couldn’t leave the house between certain times of the day and it was just not great. So I knew I had the time that I wanted to be able to do things and go out and enjoy my time at home*.”

#### 3.2.17. Sleep and Fatigue

Many participants discussed the importance of sleep and the negative impact of extreme fatigue (*n* = 22, 73%). Specifically, they noted that lack of sleep and fatigue leads to an inability to focus. As ID 28 described, “my own mental capacity, just being tired or just in a different headspace where you just kind of need to zone out on something and not be able to focus.” They also noted lack of sleep leading to less enjoyment of time with baby, as ID 1 stated “I don’t think you can enjoy a baby that is not letting you sleep.” Lastly, lack of sleep was implicated in impaired emotion regulation. Other work has presented similar findings of postpartum sleep disturbance and its resultant negative outcomes in the realms of relationships, quality of life, and emotional health [60,61,62,63].

#### 3.2.18. Social Pressures

Previous work has discussed the social pressure to engage in “intensive mothering” which can cause mothers to face judgment for not attaining a highly specific and difficult to reach standard [64]. Echoing this, many participants discussed unrealistic societal standards (as ID 18 stated “*I mean, in hindsight, I know that everything I thought about being a mom prior to becoming one was unrealistic”*), fear of social judgment for their parenting practices (see Table 2 for example quote) as well as comparing themselves to their peers and feeling insecure about their parenting performance (*n* = 18, 60%). For example, ID 24 stated, “*sometimes you see—well, you compare—it’s easy to compare with other children, and say,* “*Well, why isn’t my kid that well-behaved?”*

#### 3.2.19. Strategic Planning and Time Management

Women have historically shouldered the lion’s share of responsibility for managing the domestic sphere, and accordingly, skill with planning and organizing time as it pertains to domestic tasks has long been a lauded skill for women [65]. The participants reiterated this, frequently citing “time management” and “planning” as necessary skills for successful management of their daily responsibilities (*n* = 27, 90%). As ID 18 described, even for tasks such as spending the afternoon engaging in self-care with a friend, extensive planning and preparation was required:

“*My husband was home from work that day, so I was able to lean on him to watch the kids, but that also involved me preparing a lot of stuff to be able to leave because he’s not the typical day-to-day caregiver, so I had to make sure he had all the tools he needed to sort of keep their routine in place or keep them satisfied*.”

#### 3.2.20. Support from Others

Supported by a wealth of prior literature (see Table 2 for references), perhaps the most salient cornerstone of maternal wellbeing revealed through the interviews was the support received from others (*n* = 30, 100%). Participants discussed five key aspects of this support. The first was emotional support (i.e., having at least one person in their life who they can trust to confide in and “vent” to). The second was encouragement to engage in self-care. Participants described receiving verbal encouragement to engage in self-care, and also receiving instrumental and financial support that allowed for engagement in self-care. They characterized this encouragement as critical to their engagement in self-care. For example, several participants described their partner’s insistence that they engage in self-care activities (see Table 2 for example quote), and others described receiving offerings of childcare and financial support (e.g., money to spend at a salon, a gift card for a massage) from their social support network that made engagement in self-care more feasible. Third, participants mentioned the importance of having an engaged social network (this involved both having opportunity for social connection afforded to individuals through factors such as geographic proximity to key social support individuals, as well as having proactive outreach by social supports). Fourth, participants mentioned hands on support with childcare and home management (i.e., provision of instrumental support with childcare and/or home maintenance from a personal contact or a paid professional) as a key type of support. Lastly, participants identified partner-specific support as particularly relevant to their functioning. For those in a longterm partnership, the degree of shared responsibility for childcare and home management between partners was identified as key in determining maternal postpartum wellbeing. See Table 2 for example quotes.

#### 3.2.21. Taking Breaks and Getting Out of the House

Having the ability to be relieved from childcare duties to rest is key to a healthy postpartum adjustment [73], and participants’ reported experience supported this (*n* = 24, 80%). For example, ID 13 discussed feeling renewed and in a better headspace to spend time with her baby after receiving a break from childcare (see Table 2). Similarly, ID 2 described the way in which physical separation from the infant is key to allowing a mother to truly rest her mind from infant concerns and address her own self-care. As she stated:

“*you can’t take time for yourself completely if the baby’s around. You’re always going to be panicking… Go get some fresh air … When you’re near the baby, although you’re taking time for yourself but you’re still near the baby you’re still kind of worried about the baby*.”

Additionally, ID 29 described feeling exhausted by being constantly around her baby as:

“*I’m like her favorite person right now, so that is difficult when I’m like, ‘Just take the baby.’ And then in the morning, [my husband will] bring her downstairs, and she just wants to come back to be with me. I’m like, ‘I love you so much, but go away. I need like an hour, or like five minutes, or a breath*.’”

#### 3.2.22. Understanding Baby

Understanding the “language” of one’s newborn has been linked to a number of positive parenting outcomes [79]. Many participants echoed the importance of this skill (*n* = 25, 83%). Additionally, participants further specified the importance of “letting baby take the lead” in communication. As ID 25 stated, “you’re really watching what she’s doing, kind of giving up and allowing her to show me.” ID 8 also alluded to what experts have called “mind-mindedness”, or treating the baby as an individual with its own mind. She described utilizing “mind-mindedness” to problem-solve her infant’s difficulty sleeping in a bassinet without a swaddle: “putting myself in a baby’s shoes, thinking about what it’s like to be all snuggy, and then be like, ‘Uh, I’m just lying with my hands flying around’.”

#### 3.2.23. Workplace Flexibility and Understanding

Participants discussed the importance of understanding and flexibility from their employers (see Table 2 for example quote) and their partner’s employers (*n* = 23, 77%). More flexible and understanding workplaces made good work–life balance much more feasible, and also allowed the workplace to function as a source of emotional support for mothers. This backs up previous work which has outlined the unequal impact of parenthood on women’s employment [81,83], and how this can be psychologically straining [82].

### 3.3. Heat Map of Factor Occurrence across Domains of Functioning

**Figure 2:**
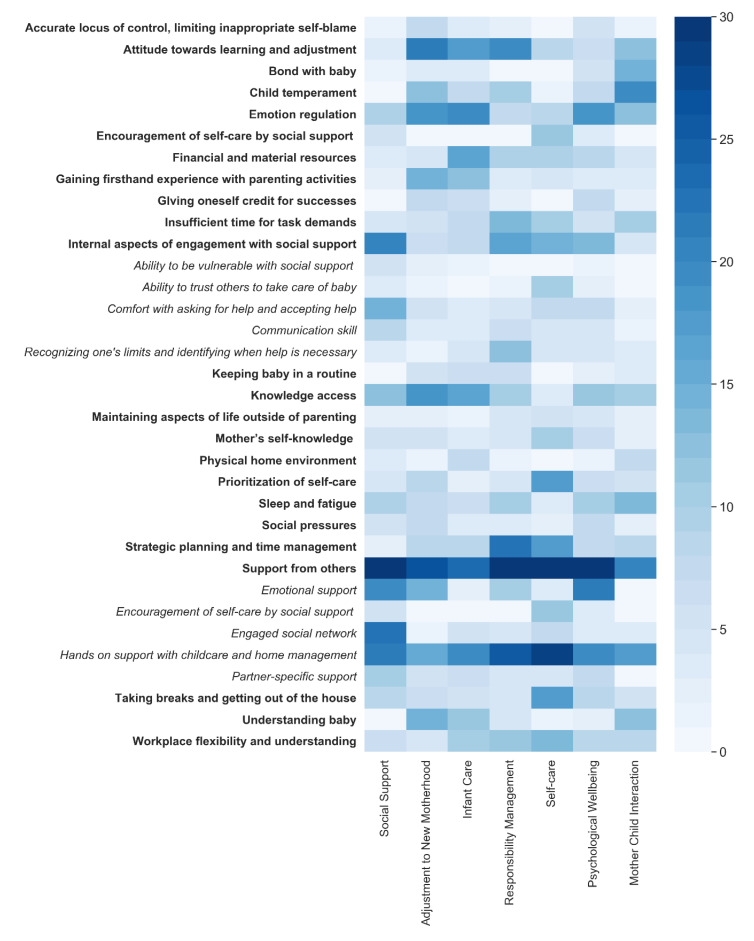
Heat map of factors across domains of functioning.

As mentioned, a heat map was created to visualize the relevance of the uncovered factors to the seven established areas of maternal functioning as indexed by number of participants who discussed each factor within each established domain of maternal functioning. Several points of consensus among the majority of participants, indicated by dark blue cell color, are evident within this figure (see Figure 2).

First is the primacy of social support, which was mentioned by nearly every participant across all domains of functioning and appears to play a critical role in perceived parenting capability. Of the subtypes of social support, hands-on or instrumental support appears particularly relevant. Every single mother brought up the importance of social support, and it was common across the domains of functioning, not solely within the domain of social support. Given the amount of work required to parent an infant, it makes sense that having another set of hands is a foundational tool for success across the domains of functioning. It is also evident from this figure that that instrumental hands-on support seemed most salient in the domain of self-care. Participants frequently stated that having someone else care for the baby was a necessary prerequisite for engaging in activities to care for themselves. Relatedly, mothers indicated that having potentially supportive individuals available was insufficient to secure the much-needed help. Rather, there were internal aspects of the participant which allowed for this support to be activated and utilized effectively. Chief among these was comfort with asking for and accepting help.

Another point of consensus was that access to knowledge was important. This knowledge took the form of both “formal” education—sources such as medical literature, classes, expert consultation (*n* = 8 mothers discussed this), as well as information from experienced others (e.g., having family or friends who have had similar experiences to the participants and, thus, have firsthand knowledge to share, discussed by *n* = 8 mothers). This knowledge access seemed to be particularly relevant for the functional domains of adjustment to new motherhood and infant care.

An additional point of consensus was that attitude towards learning and adjustment (specifically, comfort with trying new things, flexibility, and “taking the long view” and being patient with fluctuations) was an important characteristic that influenced perceived functioning. Having an adaptive attitude towards learning and adjustment seemed especially relevant in the domains of adjustment to new motherhood, infant care, and responsibility management.

Lastly, there was participant consensus concerning the importance of emotion regulation in perceived capability across several domains of functioning, including psychological wellbeing, adjustment to new motherhood, and infant care. Participants reported that the postpartum transition could be an emotionally trying time, and their ability to calm themselves in the face of challenges and stressors allowed for more enjoyable and effective interactions with their infant and a smoother adjustment overall.

## 4. Discussion

As a result of this investigation, 23 factors affecting maternal postpartum functioning were identified. All of these factors were relevant to multiple domains of maternal functioning, as is evident from the heat map (see Figure 2). Importantly, these factors are clinically actionable, and can be targeted with existing interventional approaches. In order to facilitate the process of mapping these factors to resources through which they could be addressed, a table taxonimizing these factors appears in Table 3. In the taxonomy, these factors are organized into “internal” vs. “external” factors, or those best addressed by intervening on the mother vs. those best addressed by intervening on the system surrounding the mother. The factors are then further categorized into subgroups by intervention type. This taxonomy is described further below.

With regard to individual intervention, counseling or therapy with a mental health provider may be beneficial in improving functioning. Specifically, these findings suggest postpartum mothers would benefit from mental health counseling which focuses on locus of control and limiting self-blame, adjusting their expectations and attitude towards learning and adjustment, emotion regulation, and increasing comfort with asking for help and being vulnerable. Mental health clinicians treating postpartum mothers should thus allow for a discussion of these topics. Additionally, other healthcare providers should be prepared to engage in some basic questioning about these concerns, and provide referral information for a mental health provider if it appears a mother would benefit.

Additionally, maternal functioning could be improved through the provision of education and encouragement to engage in self-care by either a clinical provider or a member of the mother’s personal social network. Specifically, an individual could ask a mother about her ability to take breaks, rest, and participate in activities outside of infant care, and provide education and encouragement surrounding these activities if met with resistance or hesitation. A clinical provider might even help a mother strategize who to seek support from, how to ask for this support, or even engage her in a role play to practice holding such a conversation.

Moreover, formal education and training could bolster functioning specifically in the areas of understanding and bonding with their baby, and initiating and maintaining routines for their baby. These resources could come in the form of a book, website, online application, through engagement in classes or peer groups, or consultation with specialists.

With regard to external factors, such as social support and access to financial and material resources, some intervention can be undertaken at the individual level. First, a healthcare provider might educate key members of a mother’s social support network on the importance of social support in the postpartum period, and encourage the provision of proactive support. In addition, mothers might be encouraged to join peer groups, which are commonly available in the community in both an in-person format (such as a class available at a local clinic or community center) or a virtual format (such as over a social media platform) as these have shown to be effective sources of support [69]. Additionally, for mothers who lack material and financial supports, healthcare providers could work to help connect postpartum mothers to existing social service resources.

However, to fully address gaps or disparities in these external factors, individual-level interventions are not sufficient; advocacy work designed to foster systemic change in financial and social support systems is also required. For example, programs currently available in other countries, such as universal nurse home visitation [87], could provide built-in social support through sending a trained specialist directly into a mother’s home. In other countries, visiting providers not only perform medical care but also individualized education and training, and may assist with tasks around the house, and this is covered by insurance [88]. Moreover, programs such as Listening Visits (a nondirective counseling intervention which originated in the United Kingdom) could be enacted in the United States to provide mothers with additional emotional support in the context of home visits [89]. Additionally, paid parental leave initiatives [88,90,91] take the onus off of mothers for balancing work and financial concerns with postpartum adjustment. Implementing similar initiatives in the United States would address critical gaps and could begin to cultivate an environment around mothers that predisposes to higher levels of postpartum function.

### Strengths, Limitations, and Future Directions

This study has several strengths. First, by conducting qualitative interviews with individuals experiencing the condition of interest (postpartum mothers), it centers the patient voice and helps to address the aforementioned gap between what experts recommend and what mothers desire in the postpartum period. Additionally, efforts were made to increase the trustworthiness of these qualitative findings. First, the coding process involved extensive immersion and discussion (as described above). Second, independent coders were utilized to reduce bias. Third, coder AA conducted all of the interviews, providing her with further insight and immersion. Lastly, the literature was consulted to provide confirmatory evidence of the salience of these findings.

However, this project also has several limitations. One limitation of this project is a potential lack of representativeness. While there was some diversity among the sample, all participants were recruited from a single large city in the Northeastern United States. Thus, there may be unique aspects to their reported experience that would not generalize to mothers of differing backgrounds. In addition, given the high level of anxiety observed in the sample, factors related to anxiety (for example, emotion regulation or anxiety as it relates to social engagement) may have been magnified in this sample in a way that does not generalize to the population at large. Moreover, it is possible that the content of the interview may have provoked anxiety and elevated the scores received on the questionnaire as it was administered following the interviews. Future work might seek to confirm the salience and consensus around these factors in other contexts. For example, interviews could be conducted in other geographic areas, or feedback on the completeness of this list of factors could be sought with a wider population of postpartum mothers through the administration of a survey across several recruitment sites (or even through an online survey, which further increases the potential sample of participants, thereby promoting generalizability).

Future work might also involve a more in-depth examination of the experience of mothers who are at higher risk for impaired functioning. This would be helpful in determining what interventional approaches might be most effective to meet their needs. Such populations might include mothers of lower SES, single mothers, mothers in the “4th Trimester” (the first three months postpartum) [92], and mothers in the “emerging adulthood” stage of development (roughly age 18–25 years) [93]. Lastly, future work could examine differences in theme generation across sociodemographic groups. This would be best achieved with a larger sample size and careful experimental design to reduce the influence of confounders.

## 5. Conclusions

A mother’s functional status during the postpartum year is of great consequence in determining the health of both mothers and their infants [13]. However, current efforts to improve functioning and adjustment during the postpartum are lacking. In particular, there appears to be a discrepancy between the opinion of experts and the opinion of mothers themselves regarding what ought to be done to help a mother thrive during this time. Therefore, to better understand what factors are most influential in determining maternal postpartum functioning, we engaged in patient-centered qualitative work with a diverse sample of postpartum mothers, and identified 23 literature-backed and clinically actionable factors that participants indicated were most influential in determining their functional capacity across multiple domains. With these data, we now have a better understanding of what mothers themselves have observed to help or hinder their postpartum function, which can be deployed in interventions to bolster maternal functioning during this critical time. Many of these factors can be addressed through individual intervention, such as providing counseling, education, and training to the mother. However, these data also indicate that advocacy to enact systemic change in the United States, such as paid parental leave initiatives and nurse home visiting programs, is necessary in order to promote ideal postpartum maternal function.

## Figures and Tables

**Table 1:** Participant sociodemographics and scores for self-report measures.

	Total (*n* = 30)	
Ethnic–Racial Background	*n* (%)	
Asian	1 (3.3)	
Biracial	1 (3.3)	
Black or African American	10 (33.3)	
Hispanic/Latinx	4 (13.3)	
Other	2 (6.7)	
White	16 (53.3)	
**Relationship Status**		
In a long-term partnership	28 (93.3)	
Single	2 (6.7)	
**Sexual Orientation**		
Bisexual	3 (10)	
Heterosexual	27 (90)	
**Partner Gender**		
Female	1 (3.3)	
Male	29 (96.6)	
**Infant Sex**		
Female	16 (53.3)	
Male	14 (46.7)	
**Parity**		
Primiparous	17 (56.7)	
Multiparous	13 (43.3)	
**Religious Identity**		
Agnostic	2 (6.7)	
Atheist	3 (10)	
Catholic	5 (16.7)	
Christian	6 (20)	
Jewish	3 (10)	
Muslim	6 (20)	
No religious/spiritual identity	1 (3.3)	
Not affiliated, but religious/spiritual	4 (13.3)	
**Education**		
4-year college graduate or Bachelor’s degree	6 (20)	
High school graduate	5 (16.7)	
Master’s Degree	8 (26.7)	
MD/PhD/JD	3 (10)	
Some college	6 (20)	
**Employment**		
Employed full time (35 h/week or more)	13 (43.3)	
Employed full time but on maternity leave, FMLA	5 (16.7)	
Employed part time (less than 35 h/week)	2 (6.7)	
Full time student	1 (3.3)	
Home maker/stay at home	3 (10)	
Unemployed―looking for work	6 (20)	
**Income**		
Less than $25,000	9 (30)	
$25,000–$49,999	3 (10)	
$50,000–$74,999	4 (13.3)	
$75,000–$99,999	1 (3.3)	
$100,000–$124,999	2 (6.7)	
$125,001–$150,000	4 (13.3)	
More than $150,000	7 (23.3)	
	***M ± SD***	**Range**
**Participant age**	30.47 ± 6.01	19–41
**Relationship length (years)**	6.27 ± 3.9	1–15
**Number of other children (for multiparous)**	1.46 ± 0.78	1–3
**Infant age (months)**	5.13 ± 3.77	0.5–12
**Participants ratings of their mother’s self-efficacy (from 1 poor to 10 very good)**	8.73 ± 2.05	3–10
	***M ± SD***	**Range**
**PSS-10 Score** ^1^	13.37 ± 7.64	1–27
**BIMF** ^2^	97.9 ± 10.66	70–116
**HADS-A** ^3^	6.73 ± 4.36	0–18
**HADS-D** ^3^	3.43 ± 2.92	0–10

^1^ The population norm is a score of 13.7. ^2^ Scores to the right of 80 on a number line indicate ideal functioning. ^3^ Scores above 7 on either subscale indicate clinically elevated symptoms.

**Table 2:** Factors influencing maternal functioning revealed through qualitative interviews.

Factor	*n* (Mothers Who Discussed Factor)	Supporting Literature	Example Quotes:
Accurate locus of control, limiting inappropriate self-blame	12	[24,25]	*“And just sort of developmental milestones in your kid, too, and feeling like things are on pace. Because it’s hard to disaggregate what you’re doing from what you’re seeing even though not―not that we’re irrelevant, but not everything that you see in your child is a direct result of things that you’ve done.”*
Attitude towards learning and adjustment	28	[26,27,28,29]	*“Knowing that, yeah, I can do this. It’s going to be a pain in the butt. It sounds like a lot of things, but what am I going to do? Never drive in the car with her? It’s something I have to do, so put on your big girl pants and let’s do this.”* *“Flexibility. It changes almost daily sometimes and you have to be able to go with the flow.”* *“You might have shitty days, but it gets better.”*
Bond with baby	16	[30,31]	*“Some people actually go somewhere else with their baby just to have their one-on-one time. Me, nobody come in my room. Close the door, lock it, and I need some time. I shoo everybody away. Even the phone. I’ll put it on vibrate. I don’t want to hear it ringing, so I can pay attention … My determination to have my bonding time because I feel like babies grow so fast. It’s a month already and it felt like yesterday. I have to be present if I don’t want to miss anything. So I have to learn him, his cues, what faces he make, what they mean. There’s certain cries. He has a certain cry for certain things … I have to learn all this, so I need to pay attention. So I need my time, so I’m determined.”*
Child temperament	27	[32]	*“I mean we’ve had I’d say only on three occasions but they were traumatic and embedded in our memory where she cried for like 45 min straight and I mean it’s so wearing. It’s the worst thing … like what you use to torture people.”*
Emotion regulation	29	[33,34]	*“I think in preparation for parenthood, I got into mindfulness and meditation, and so that really helped me sort of find space and calmness and ways to just breathe through any stressors or tension, and sort of reassure myself that all the moments are manageable.”*
Financial and material resources	21	[23,35,36]	*“I mean, I think the biggest is resources financially and help because it’s like I don’t know how people who don’t have any help or don’t have the financial means to get the help could even attempt to take care of the kids and take care of themselves and the household and if they work … I just think that’s the biggest thing.”*
Gaining firsthand experience with parenting activities	24	[37,38,39]	*“Just the experience of taking care of the kids and it comes naturally … each child is different, but you learn more as you work with them.”*
Giving oneself credit for successes	16	[40,41]	*“It’s hard for people to give themselves positive feedback. So when I do have a meeting or hangout, so with one or two moms, you don’t hear as many positive statements about, “Oh, I’m doing a great job.”* *“So give yourself some grace and knowing by yourself that you’re doing a good job, even if you don’t feel it at the time.”*
Insufficient time for task demands	26	[42]	*“Lack of time. Trying to pump through times during the day with―we have a very set schedule as a physical therapist. So it’s like you’ll see a patient here, here, and here. You have an hour break, an hour break, and when you’re spending 20 min, there’s no time for that sometimes. And I’m very social, so. I feel like I have to shut myself down sometimes to get everything done.”*
Internal aspects of engagement with social support	27 (totals aggregated from subthemes)		
*Ability to be vulnerable with social support*	7	[43]	*“Just being really open. Almost grossly open of describing what’s going on with your body and just stuff like that, so... Or being able to open up, just about my self-doubt and those things that I had been feeling that kind of felt dark or they’re not the most pleasant thing to talk to people about, but being able to do that.”*
*Ability to trust others to take care of baby*	15	[44]	*“Having somebody that you trust to leave the baby is very important because then you’re not stressing out the whole time about like, ‘Oh, what the baby is going to do?’ It’s not crying the whole time. So that’s really the most important thing.”*
*Comfort with asking for help and accepting help*	20	[43,45,46,47]	*“Sometimes I don’t reach out when I should and that could probably get in the way. Because then, I have the extra help but then I don’t reach out for it. And then, I start to get frustrated and not being able to take care of myself. And then, the emotions start to come … because sometimes I feel like I can do everything on my own and then I can’t.”*
*Communication skill*	15	[48,49,50]	*“Most definitely communication, because as long as you got somebody next to you and you can communicate, ‘Listen, I need your help because I need to adjust a little bit more. I need you to help me get this schedule together, I need you to help me do this because …’ you just have to talk about it and you have to get it out. You have to.”*
*Recognizing one’s limits and identifying when help is necessary*	16	[27,39]	*“Sometimes you get in your own way by saying I can do everything and then some and then it’s just way too much.”*
Keeping baby in a routine	14	[51]	*“I think the knowing that both of my children are content, and we’re prepared for the next day, and knowing that they’re feeling well, and I’m not anticipating disrupted sleep or those sort of things which, I think, I have been able to really establish through a really good routine with them. So I’m able to sort of feel that calm at the end of the day.”*
Knowledge access	26	[52,53,54]	*“My working knowledge of being able to take care of my baby, and having the resources if I needed it with lactation or being able to call my pediatrician if anything is wrong. And then also just having my family there as backup just in case. Because they’ve had babies, so they kind of have experience in certain things.”*
Maintaining aspects of life outside of parenting	14	[55,56]	*“I would say don’t cancel out all the things that you were doing before because I know you’re busy, and you have a newborn, and you’re trying to focus on it, but try to remember some of the things that you used to like doing before you had the baby. If it’s listening to music or reading a book or something like that because my husband, he literally told me, ‘I wish you would go out with your friends [laughter].’ Because during the nine months, I stayed by myself, and now that I had the baby, I’m still doing it. And he was like, ‘No, it’s not good for you. You need to go out and socialize with people.’ So try not to close off because when you close off from people, you don’t notice it, but your moods change when you get around people.”*
Mother’s self-knowledge	20	[57]	*“I guess just knowing yourself too, and knowing what do you need to do to keep yourself mentally in a good place.”*
Physical home environment	15	[58]	*“I can say that I have what I have, but it’s not stable. It’s not a stable situation. So it’s like, let’s say―I can be in this situation that I can be kicked out today or tomorrow and I won’t have nowhere. I’ll have to figure that out like fast. And that’s something that I don’t ever want to happen.”*
Prioritization of self-care	24	[59]	*“On one side, you feel that it feels good because finally, after a long time, then you’re doing something for yourself, and you know that it’s good for the baby as well because it might help you to get happier and less stressed. And on the other side, then you kind of feel guilty because you’re just taking time away from your baby.”*
Sleep and fatigue	22	[60,61,62,63]	*“Energy, which … There’s only so much stamina that one person can sustain, right? Before it’s, you’re just too tired. And I find in life, there aren’t hours to do it. There’s just literally sometimes—a depleted resource or energy. Where you’re just, ‘all I can do is sit on this couch.’ And I feel like there’s so many things I could be doing that are among my life goals, but I just cannot make my brain want to do those things right now.”*
Social pressures	18	[64]	*“We just recently, for example, just switched her out of her bassinet. And I didn’t want to talk to anybody about it. Because I felt like we had already left her in there. Our in-laws were complaining that she’s too big for it like a month ago. She’s fine. I think it worked. And then I didn’t want to tell anybody that we were using. I was afraid what they’re going to say.”*
Strategic planning and time management	27	[65]	*“I’m also very organized. I mean, I’ve always been a planner, but I would say I’m more so organized now, keeping lists, things like that; things that I need to do on a daily, weekly basis; maintaining schedules for the girls in the morning, afternoon, evening, specifically bedtime now.”*
Support from others	30 (totals aggregated from subthemes)		
*Emotional support*	28	[66,67,68,69]	*“Yeah. I think someone to talk to is the biggest one. Because I just―I don’t know. I had so many doubts and whether I was doing the right thing or not or trying to figure out the right thing to do for a lot of different scenarios. So yeah, just helped me to be able to talk about that to somebody.”*
*Encouragement of mother’s self-care by social support*	15	[70,71]	*“Getting out. Going to a yoga class once a week. I would just come home a totally different person. I’m very frugal and I don’t spend money on those things and my husband was like, “You need to go to that every week. You just come home refreshed … it’s good for you.”*
*Engaged social network*	26	[45,72]	*“My mom. She’s just a helpful person. And she has six grandkids so she’s just like, ‘Oh, do you need any help? I’m coming over. I’m going to help you do this,’ I’m like, ‘Okay.’ I’m like, ‘That is fine.’ I’m like, ‘I don’t get to worry about all this alone anymore.’ And she just helped me out. She’s a big help.”*
*Hands on support with childcare and home management*	30	[45,66,73]	*“In my opinion, the most important thing is other human resources. So whether it’s a partner or someone else who’s available to do those things. Because if you can’t have someone else taking care of your child, you can’t do anything separate from your child to take care of yourself. So I think that’s huge, and just from some talks to other moms, people who are really struggling, it often seems like that was the tension. Like a partner who works a lot, or family that is far away … no matter how capable you are, at some point you just can’t, and you need someone else to change a diaper or get up in the middle of the night, feed a bottle. You just can’t … And people who don’t have as much of that really end up at their wits’ end.”*
*Partner-specific support*	18	[74,75,76,77,78]	*“For me, it’s just that my partner is just awesome, and he’s just super supportive … So, yeah. He knows that we’re a team, and he’s very appreciative of the parent that I am and everything.”*
Taking breaks and getting out of the house	24	[73]	*“My mother-in-law takes the baby in the morning and then I get her back at 12:00 noon, then she’s happy, she’s fed, she’s changed and she took a good nap too. So then when she comes back, she’s all good and I’m able to have that time with her where I’m not like, ‘Oh, why don’t you go to sleep? I fed you an hour ago and now, you want me to feed you again’, and stuff like that.”*
Understanding baby	25	[79,80]	*“I know when he’s tired. I know when he’s mean. I know when he don’t want to be bothered. I just know. I know my son. My daughter will tell me in a minute that there’s nothing wrong with her, but I know when something’s wrong with her. So I know my children very well. That’s why I communicate with them in the way that I do.”*
Workplace flexibility and understanding	23	[81,82,83]	*“So I think on the work side, it’s very important to have an environment that is supportive. So your boss and the organization you’re working for, being supported is very important. Because in the beginning, you’re just trying to figure out this new motherhood thing, so*―*having somebody that supports you really helps you, not just practically but also psychologically.”*

**Table 3:** Intervention taxonomy mapping the factors impacting maternal functioning to interventions through which they may be addressed.

“Internal” Factors, Best Addressed by Intervening on the Mother	“External” Factors, Best Addressed by Intervening on the System Surrounding the Mother
**Mental Health Counseling:** ✓ Accurate locus of control, limiting inappropriate self-blame ✓ Adaptive attitude towards learning and adjustment ✓ Emotion regulation ✓ Giving oneself credit for success ✓ Internal aspects of engagement with social support ⚬ Ability to be vulnerable ⚬ Ability to trust others to take care of baby ⚬ Comfort with asking for help/accepting help ⚬ Communication skill ⚬ Recognizing limits ✓ Self-knowledge ✓ Social pressures	**Encouragement of Mother’s Personal Social Support Network, Connecting Mother to Other Sources of Support (e.g., mom’s groups), Advocacy:** ✓ Support from others ⚬ Emotional support ⚬ Encouragement of self-care ⚬ Engaged social network ⚬ Hands on support with childcare and home management ⚬ Partner-specific support **Discussion, Education, and Encouragement of Employer, Potential Outreach to Social Services, Advocacy:** ✓ Financial and material resources ✓ Insufficient time for task demands ✓ Physical home environment ✓ Workplace flexibility and understanding
**Encouragement and Education of Mother About the Importance of Self-Care:**	
✓ Maintaining aspects of life outside of parenting	
✓ Prioritization of self-care	
✓ Taking breaks	
✓ Sleep and fatigue	
**Formal Educational Resources and/or Training:**	
✓ Bonding with baby	
✓ Child temperament	
✓ Gaining firsthand experience with parenting	
✓ Keeping baby in a routine	
✓ Knowledge access	
✓ Strategic planning and time management	
✓ Understanding baby	

## Appendix A

Below is the interview guide used to conduct the structured interviews.

### Appendix A.1. Domain: Infant Care

Have you heard the term “infant care”? To make sure we’re on the same page, when I say that, I mean: taking care of the day-to-day caretaking tasks for your baby (feeding, cleaning, etc.)

1. a. Take a moment and think about the “average” mom, not yourself. In order for that “average” mom to take care of her baby well, what are the three most important things she needs? These could be skills, resources, or anything else that a mom might need to successfully take care of her baby on a regular basis?

2. a. Take a moment and think of a time or a specific example of when you felt you successfully took care of your baby. In 1–2 sentences, briefly tell me what that looked like?

b. In that specific example you just gave about infant care, what things made it work out so well?

c. Take another moment and think of a time when you did not take care of your baby’s needs as well, even though you tried to. In 1–2 sentences, what did that look like?

d. In that specific example of infant care you just gave, what got in the way of it working out?

3. a. When thinking about your own life now, what about yourself or your environment allows you to take care of your infant successfully? What about yourself or your environment get in the way of taking care of your infant?

### Appendix A.2. Domain: Mother–Child Interaction

Have you heard the term “mother–child interaction”? To make sure we’re on the same page, when I say that, I mean: having a relaxing and enjoyable time with your baby.

1. a. Take a moment and think about the “average” mom, not yourself. In order for that “average” mom to have a relaxing and enjoyable time with her baby, what are the three most important things she needs? These could be skills, resources, or anything else that a mom might need to successfully have a relaxing and enjoyable time with her baby on a regular basis?

2. a. Take a moment and think of a time or a specific example of when you successfully had a relaxing and enjoyable time with your baby. In 1–2 sentences, briefly tell me what that looked like?

b. In that specific example you just gave about having a relaxing and enjoyable time with your baby, what do you think helped to make that happen?

c. Take another moment and think of a time when you were not able to have a relaxing and enjoyable time with your baby despite trying to. In 1–2 sentences, what did that look like?

d. In that specific example of not having a relaxing and enjoyable time even though you tried to, what got in the way of it working out?

3. a. When thinking about your own life now, what about yourself or your environment allow for you to successfully have a relaxing and enjoyable time with your baby? What about yourself or your environment get in the way of having a relaxing and enjoyable time?

### Appendix A.3. Domain: Psychological Wellbeing

Have you heard the term “psychological wellbeing”? To make sure we’re on the same page, when I say that, I mean: living with little distress, anxiety, or mood issues, feeling in a relatively “good place” emotionally.

1. a. Take a moment and think about the “average” mom, not yourself. In order for that “average” mom to be in a good place emotionally, what are the three most important things she needs? These could be skills, resources, or anything else that a mom might need to be in a good place emotionally on a regular basis?

2. a. Take a moment and think of a time or a specific example of when you were able to be in a good place emotionally as a mom. In 1–2 sentences, briefly tell me what that looked like?

b. In that specific example you just gave about being in a good place emotionally, what things made it successful?

c. Take another moment and think of a time when you were not able to be in a good place emotionally as a mom even though you tried to. In 1–2 sentences, what did that look like?

d. In that specific example of not being in a good place emotionally you just gave, what got in the way?

3. a. When thinking about your own life now, what about yourself or your environment allow for you to be a in a good place emotionally? What about yourself or your environment get in the way of being in a good place emotionally?

### Appendix A.4. Domain: Social Support

Have you heard the term “social support”? To make sure we’re on the same page, when I say that, I mean: getting support or help from other individual’s in a mom’s life.

1. a. Take a moment and think about the “average” mom, not yourself. In order for that “average” mom to feel socially supported, what are the three most important things she needs? These could be skills, resources, or anything else that a mom might need to feel social support on a regular basis?

2. a. Take another moment and think of a time or a specific example of when you were able to feel socially supported as a mom. In 1–2 sentences, briefly tell me what that looked like?

b. In that specific example you just gave about a time you felt socially supported, what things made it successful?

c. Take another moment and think of a time when you did not feel socially supported, even if you tried to. In 1–2 sentences, what did that look like?

d. In that specific example you just gave, what got in the way of you feeling socially supported?

3. a. When thinking about your own life now, what about yourself or your environment help you to feel socially supported? What about yourself or your environment get in the way of you feeling socially supported as a mom?

### Appendix A.5. Domain: Responsibility Management

Have you heard the term “responsibility management”? To make sure we’re on the same page, when I say that, I mean: being able to balance and complete other responsibilities in addition to baby ones.

1. a. Take a moment and think about the “average” mom, not yourself. In order for that “average” mom to successfully balance and complete her baby responsibilities and her other life responsibilities, what are the three most important things she needs? These could be skills, resources, or anything else that a mom might need to successfully balance and complete your baby responsibilities and your other life responsibilities on a regular basis?

2. a. Take a moment and think of a time or a specific example of when you were able to successfully balance and complete your baby responsibilities and her other life responsibilities. In 1–2 sentences, briefly tell me what that looked like?

b. In that specific example you just gave about balancing and completing your baby responsibilities and her other life responsibilities, what things made it successful?

c. Take another moment and think of a time when you were not able to balance and complete your baby responsibilities and her other life responsibilities even though you tried to. In 1–2 sentences, what did that look like?

d. In that specific example you just gave, what got in the way?

3. a. When thinking about your own life now, what about yourself or your environment allow for successful balance and completion of your baby responsibilities and her other life responsibilities? What about yourself or your environment get in the way of balancing and completing your baby and other responsibilities?

### Appendix A.6. Domain: Adjustment to New Motherhood

Have you heard the term “adjustment to new motherhood”? To make sure we’re on the same page, when I say that, I mean: over time feeling like you are getting better and more used to taking care of your baby.

1. a. Take a moment and think about the “average” mom, not yourself. In order for that “average” mom to feel better and more used to taking care of her baby, what are the three most important things she needs? These could be skills, resources, or anything else that a mom might need to feel adjusted on a regular basis?

2. a. Take a moment and think of a time or a specific example of when you were able to achieve a feeling like she is getting better and more used to taking care of her baby. In 1–2 sentences, briefly tell me what that looked like?

b. In that specific example you just gave, what helped it happen?

c. Take another moment and think of a time when you did not feel like you were getting better and more used to taking care of your baby. In 1–2 sentences, what did that look like?

d. In that specific example you just gave, what got in the way?

3. a. When thinking about your own life now, what about yourself or your environment help you to feel better and more able to take care of your baby? What about yourself or your environment get in the way?

### Appendix A.7. Domain: Self-Care

Are you familiar with the topic of “self-care”? To make sure we’re on the same page, when I say that, I mean taking time for yourself.

1. a. Take a moment and think about the “average” mom, not yourself. In order for that “average” mom to take time for herself, what are the three most important things they need? These could be skills, resources, or anything else that a mom might need to be able to take time for herself on a regular basis?

2. a. Take another moment and think of a time or a specific example of when you did a successfully took time for yourself as a mom. In a 1–2 sentences, briefly tell me what that looked like?

b. In that specific example you just gave about taking time for yourself, what things made it successful, what made it happen?

c. Take another moment and think of a time when you tried to take time for yourself, but it didn’t work out. In a 1–2 sentences, what did that look like?

d. In that specific example of a time that you weren’t able to take time for yourself what got in the way?

3. a. When thinking about your life now, what are some things about yourself or your environment allow for you to take time for yourself successfully? What about yourself or your environment get in the way of you taking time for yourself?
